# Supplementary material for: Comparative Pathogenomic Analysis of Two Banana Pathogenic Dickeya Strains Isolated from China and the Philippines
Source: Int J Mol Sci. 2022 Oct 22;23(21):12758. doi: 10.3390/ijms232112758 (PMC9653667; doi:10.3390/ijms232112758)
Supplement: Supplementary file 1 [file ijms-23-12758-s001.zip › ijms-1934934-supplementary.pdf]

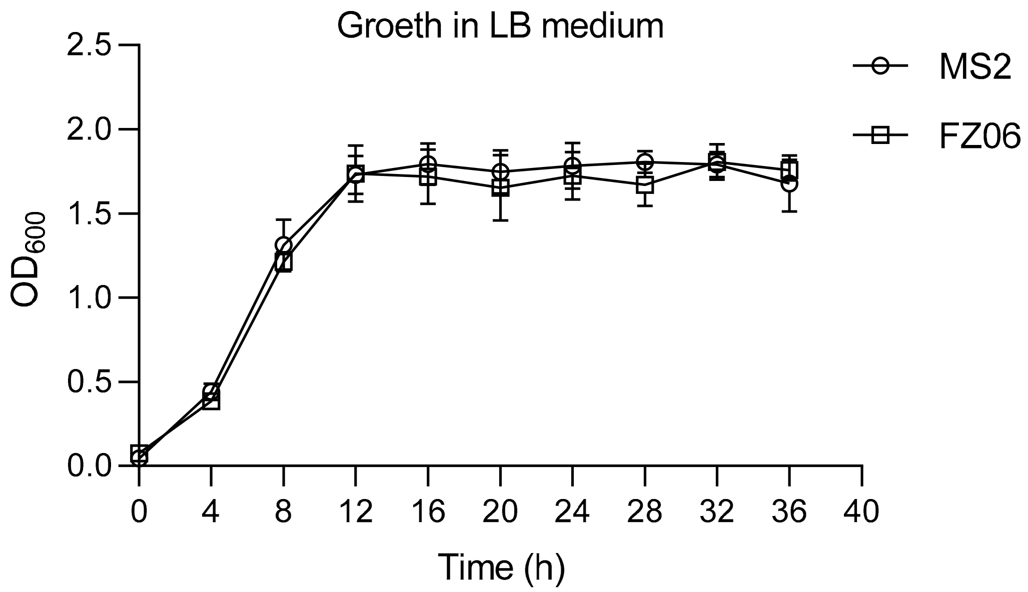

Figure S1. Growth curves of strains FZ06 and MS2 in LB medium.

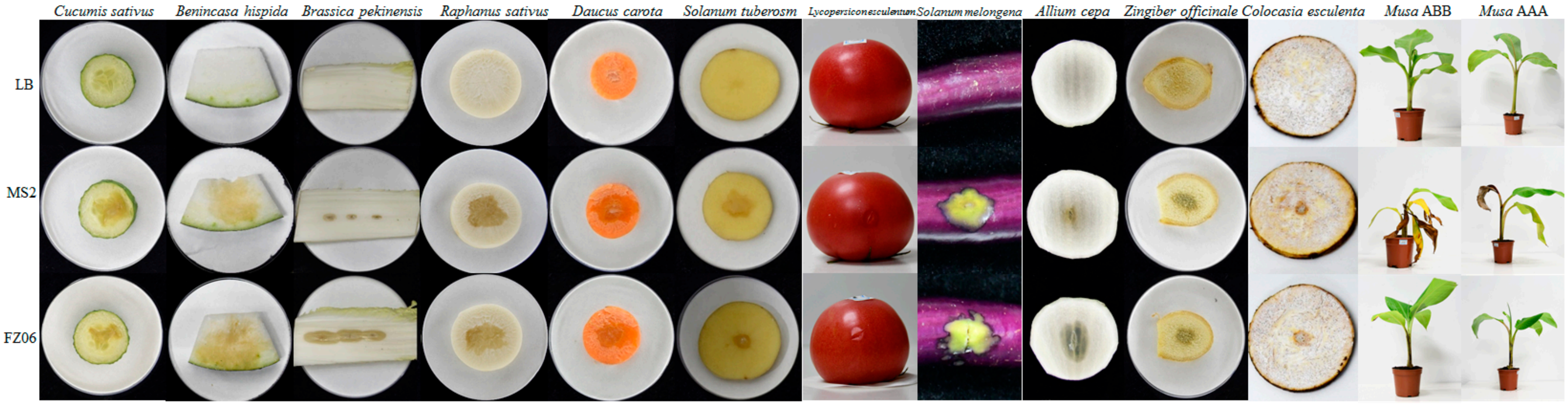

Figure S2. Disease symptoms following infection by strains FZ06 and MS2 in various dicot and monocot hosts.

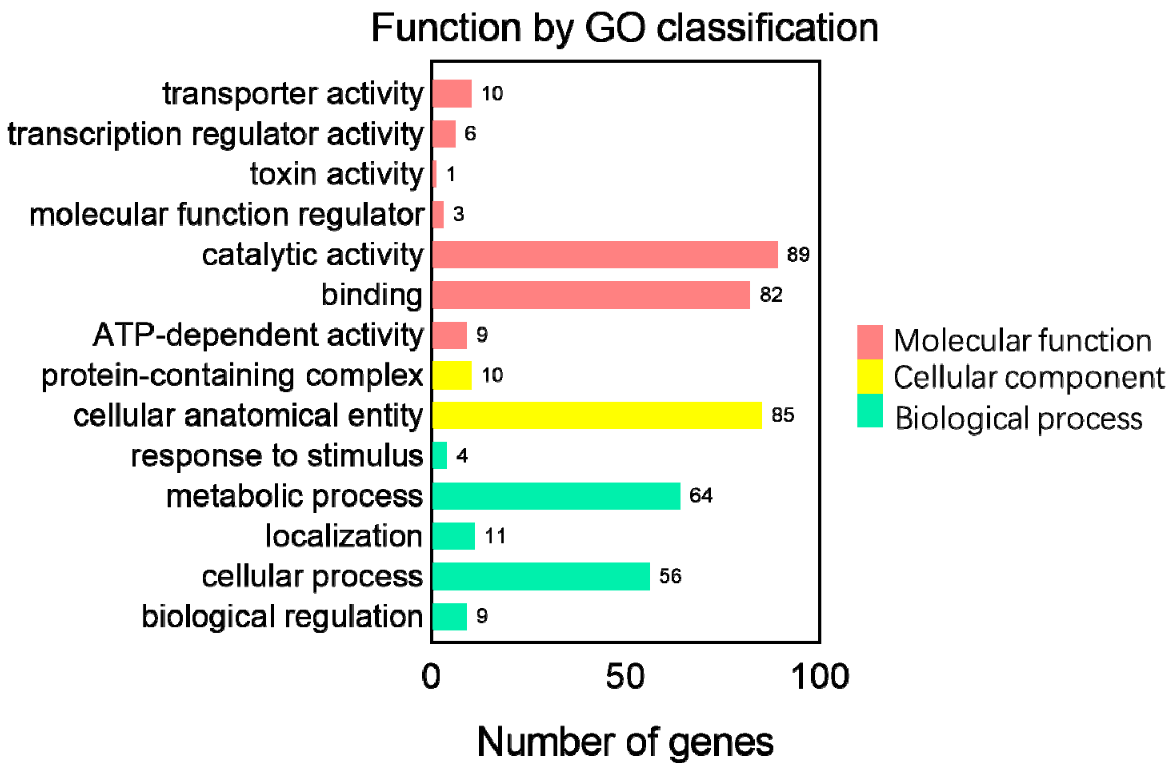

Figure S3. GO function annotation of specific genes within strain FZ06.

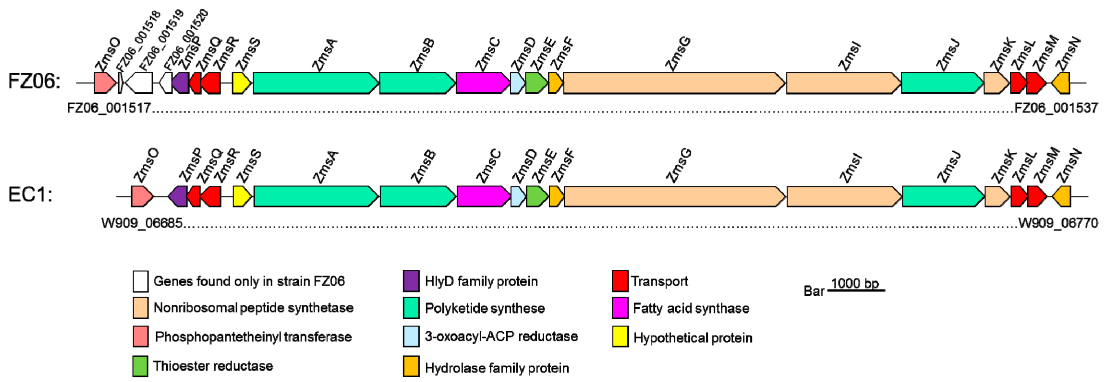

Figure S4. Genomic organization of the zeamine biosynthetic gene clusters in strains FZ06 and EC1.

Table S1. The Average Nucleotide Identity (ANI) values between FZ06 genome and the *Dickeya* genomes in NCBI database

Species names	Subject genome	ANI value (%)	Species names	Subject genome	ANI value (%)
Dickeya *dadantii* A622-S1-A17	GCF_020406675.1	98.4955	*Dickeya dianthicola* 16MB01	GCF_018361125.1	92.1453
Dickeya dadantii S3-1	GCF_018904205.1	98.2915	*Dickeya dianthicola* DDI_16NJ12	GCF_018596235.1	92.1421
Dickeya dadantii NCPPB 2976	GCF_000406185.1	96.9039	*Dickeya dianthicola* DDI_16PA07	GCF_018596285.1	92.1107
Dickeya dadantii CZ1501	GCF_001995275.1	96.5302	*Dickeya dianthicola* CFBP2982	GCF_009873565.1	92.1097
Dickeya dadantii Yana2-2	GCF_013168455.1	96.5103	*Dickeya dianthicola* WV516	GCF_002906735.1	92.0941
Dickeya dadantii M2-3	GCF_020047155.1	96.4982	*Dickeya dianthicola* MIE34	GCF_009874285.1	92.092
Dickeya dadantii BI3-1	GCF_009903315.1	96.4864	*Dickeya dianthicola* NY1785A	GCF_016106715.1	92.0877
Dickeya dadantii Housui2-1	GCF_013168465.1	96.48	*Dickeya dianthicola* DDI_59W	GCF_018596255.1	92.0849
Dickeya dadantii 3937	GCF_000147055.1	96.4695	*Dickeya dianthicola* NY1547B	GCF_016106895.1	92.084
Dickeya dadantii NCPPB 3537	GCF_000406265.1	96.467	*Dickeya dianthicola* NY1559C	GCF_016106815.1	92.079
Dickeya dadantii Kousui1-1	GCF_013168475.1	96.423	*Dickeya dianthicola* SS70	GCF_003121805.1	92.0768
Dickeya dadantii BI1-1	GCF_013168495.1	96.4182	*Dickeya dianthicola* NY1538B	GCF_016106925.1	92.0768
Dickeya dadantii Kunimi-3	GCF_013168575.1	96.4173	*Dickeya dianthicola* DE440	GCF_002906725.1	92.0727
Dickeya dadantii DSM 18020	GCF_003049785.1	96.3867	*Dickeya dianthicola* DDI_16NJ11	GCF_018596245.1	92.0723
Dickeya dadantii Aka1-1	GCF_013168485.1	96.341	*Dickeya dianthicola* GBBC 2039	GCF_000365365.1	92.0676
Dickeya dadantii NCPPB 898	GCF_000406145.1	96.3092	*Dickeya dianthicola* NY1556C	GCF_016106945.1	92.0649
Dickeya solani IFB0311	GCF_013339815.1	94.4315	*Dickeya dianthicola* CFBP2015	GCF_009873535.1	92.0638
Dickeya solani D s0432-1	GCF_002846975.1	94.4309	*Dickeya dianthicola* NY1528B	GCF_016106995.1	92.0617
Dickeya solani IFB0167	GCF_013334125.1	94.4252	*Dickeya dianthicola* NY1557A	GCF_016106935.1	92.0614
Dickeya solani D s0432-1	GCF_000474655.1	94.4208	*Dickeya dianthicola* NY1746A	GCF_016106695.1	92.0598
Dickeya solani IFB0487	GCA_013339845.1	94.4139	*Dickeya dianthicola* NY1558D	GCF_016106885.1	92.0576
Dickeya solani IFB0231	GCF_013334145.1	94.4134	*Dickeya dianthicola* NY1760A	GCF_016106685.1	92.0555

Dickeya solani IPO2019	GCF_017161585.1	94.4126	*Dickeya dianthicola* S4.16.03.LID	GCF_003595005.1	92.0538
Dickeya solani IFB0223	GCF_003718335.1	94.4104	*Dickeya dianthicola* NY1758A	GCF_016106725.1	92.0533
Dickeya solani D12	GCF_014751545.1	94.4098	*Dickeya dianthicola* S4.16.03.P2.4	GCF_003595075.1	92.0506
Dickeya solani CH05026	GCF_016404895.1	94.4094	*Dickeya dianthicola* RNS04.9	GCF_000975305.1	92.0501
Dickeya solani IFB0212	GCF_013339795.1	94.4064	*Dickeya dianthicola* NCPPB 453	GCF_000365305.1	92.0498
Dickeya solani RNS10-27-2A	GCF_009874415.1	94.4036	*Dickeya dianthicola* NY1536B	GCF_016106985.1	92.0484
Dickeya solani F012	GCF_002921195.1	94.4026	*Dickeya dianthicola* NCPPB 3534	GCF_000365405.2	92.0443
Dickeya solani F012	GCF_002924265.1	94.4026	*Dickeya dianthicola* NY1578A	GCF_016106795.1	92.021
Dickeya solani IFB0421	GCF_013334185.1	94.4013	*Dickeya dianthicola* RNS04.9	GCF_002706485.1	92.0153
Dickeya solani RNS 08.23.3.1. A	GCF_000511285.2	94.4001	*Dickeya dianthicola* NY1562C	GCF_016106855.1	92.0115
Dickeya solani IFB0417	GCA_013334165.1	94.3988	*Dickeya dianthicola* A260-S21-A16	GCF_020406895.1	92.0011
Dickeya solani IFB 0099	GCF_000831935.2	94.3967	*Dickeya dianthicola* NY1719A	GCF_016106785.1	92.0008
Dickeya solani IPO 2222	GCF_001644705.1	94.3946	*Dickeya dianthicola* NY1713C	GCF_016106805.1	91.9873
Dickeya solani IFB0695	GCF_013339875.1	94.3934	*Dickeya dianthicola* CFBP1888	GCF_009873515.1	91.9774
Dickeya solani IFB_0221	GCF_003600975.1	94.393	*Dickeya dianthicola* IPO 980	GCF_000430955.1	91.9627
Dickeya solani PPO 9019	GCF_002846995.1	94.392	*Dickeya dianthicola* RNS 11-47-1-1A	GCF_009874325.1	91.9521
Dickeya solani MK10	GCF_000365285.1	94.3884	*Dickeya undicola* 2B12	GCF_000784735.1	89.604
Dickeya solani A623-S20-A17	GCF_020406975.1	94.3858	*Dickeya undicola* FVG1-MFV-O17	GCF_003725615.1	89.4924
Dickeya solani CH9918-774	GCF_016404885.1	94.3855	*Dickeya undicola* FVG10-MFV-A16	GCF_003725575.1	89.4325
Dickeya solani Am3a	GCF_009874395.1	94.385	*Dickeya chrysanthemi* Ech1591	GCF_000023565.1	87.4373
Dickeya solani RNS 07.7.3B	GCF_001401695.1	94.3842	*Dickeya chrysanthemi* NCPPB 402	GCF_000406105.1	87.4294
Dickeya solani Sp1a	GCF_009874405.1	94.3833	*Dickeya chrysanthemi* EC16	GCF_019739115.1	87.4166
Dickeya solani MIE35	GCF_009874305.1	94.3808	*Dickeya chrysanthemi* L11	GCF_000784725.1	87.3986
Dickeya solani CH07044	GCF_016404925.1	94.3794	*Dickeya chrysanthemi* NCPPB 3533	GCF_000406245.1	87.3746
Dickeya solani IFB_0158	GCF_003600995.1	94.3759	*Dickeya* sp. Ws52	GCF_007210685.1	87.3469

Dickeya solani PPO 9019	GCF_001506125.1	94.374	*Dickeya chrysanthemi* A604-S21-A17	GCF_020406775.1	87.279
Dickeya solani MK16	GCF_000365345.1	94.3695	*Dickeya chrysanthemi* NCPPB 516	GCF_000406065.1	87.2379
Dickeya solani RNS 05.1.2A	GCF_001401705.1	94.3692	*Dickeya poaceiphila* NCPPB 569	GCF_000406125.1	86.3227
Dickeya solani M21a	GCF_009873555.1	94.3675	*Dickeya* sp. CFBP 2040	GCF_012273755.1	86.3104
Dickeya solani IPO 2222	GCF_000400795.1	94.364	*Dickeya poaceiphila* NCPPB 569	GCF_007858975.2	86.2847
Dickeya solani CH9635-1	GCF_016404945.1	94.3628	*Dickeya zeae* PL65	GCF_019464635.1	85.7813
Dickeya solani GBBC 2040	GCF_000400565.1	94.3498	*Dickeya zeae* JZL7	GCF_019444095.1	85.7523
Dickeya solani PPO 9134	GCF_001417915.1	94.3468	*Dickeya zeae* CE1	GCF_012278555.1	85.718
Dickeya fangzhongdai ND14b	GCF_000758345.1	92.8183	*Dickeya zeae* MS2	GCF_002887555.1	85.5878
Dickeya fangzhongdai AP6	GCF_009873225.2	92.7633	*Dickeya zeae* MS1	GCF_000382585.2	85.5707
Dickeya fangzhongdai CGMCC 1.15464	GCF_014642955.1	92.7526	*Dickeya parazeae* Ech586	GCF_000025065.1	85.565
Dickeya fangzhongdai B16	GCF_001187975.3	92.7447	*Dickeya zeae* A586-S18-A17	GCF_020520245.1	85.5549
Dickeya fangzhongdai 908C	GCF_017656535.1	92.7437	*Dickeya zeae* MS_2014	GCF_021614775.1	85.527
Dickeya fangzhongdai QZH3	GCF_014854775.1	92.7412	*Dickeya zeae* A661-S21-A17	GCF_020406575.1	85.5167
Dickeya fangzhongdai PA1	GCF_003628775.1	92.7355	*Dickeya zeae* MS_2018	GCF_021614795.1	85.5122
Dickeya fangzhongdai LN1	GCF_014854755.1	92.7337	*Dickeya zeae* MK19	GCF_000406325.1	85.4667
Dickeya fangzhongdai DSM 101947	GCF_002812485.1	92.729	*Dickeya parazeae* S31	GCF_017897605.1	85.4565
Dickeya sp. Secpp 1600	GCF_003049805.1	92.7279	*Dickeya zeae* A5410	GCA_019464615.1	85.45
Dickeya fangzhongdai M005	GCF_000803195.1	92.7241	*Dickeya zeae* NCPPB 2538	GCF_000406165.1	85.4346
Dickeya fangzhongdai M074	GCF_000774065.1	92.7153	*Dickeya zeae* EC1	GCF_000816045.1	85.4174
Dickeya sp. MK7	GCF_000406305.1	92.7009	*Dickeya zeae* NCPPB 3532	GCF_000400525.1	85.4158
Dickeya fangzhongdai S1	GCF_001187965.2	92.6441	*Dickeya zeae* ZJU1202	GCF_000264075.1	85.4005

Dickeya fangzhongdai 643b	GCF_022385255.1	92.6345	*Dickeya zeae* EC2	GCF_012278405.1	85.3842
Dickeya sp. NCPPB 3274	GCF_000406205.1	92.6011	*Dickeya oryzae* S20	GCF_017897305.1	85.3453
Dickeya dianthicola 67-19	GCF_014893095.1	92.2965	*Dickeya oryzae* A003-S1-M15	GCF_020406815.1	85.321
Dickeya dianthicola 16JP05	GCF_018361225.1	92.1644	*Dickeya oryzae* FVG03	GCF_017897245.1	85.3042
Dickeya dianthicola ME23	GCF_003403135.1	92.1643	*Dickeya oryzae* FVG08	GCF_017897585.1	85.2964
Dickeya dianthicola PA24	GCF_018361045.1	92.1643	*Dickeya zeae* DZ2Q	GCF_000404105.1	85.2907
Dickeya dianthicola 16SBJ16	GCF_018361065.1	92.1643	*Dickeya oryzae* CSL RW192	GCF_000406045.1	85.2895
Dickeya dianthicola 16ME22T	GCF_018361085.1	92.1643	*Dickeya zeae* NCPPB 3531	GCF_000406225.1	85.2735
Dickeya dianthicola 16ME21T	GCF_018361105.1	92.1643	*Dickeya oryzae* ZYY5	GCF_009372235.1	85.2716
Dickeya dianthicola 16MA15T	GCF_018361145.1	92.1643	*Dickeya oryzae* A642-S2-A17	GCF_020406685.1	85.1972
Dickeya dianthicola 16LI02	GCF_018361185.1	92.1643	*Dickeya aquatica* 174/2	GCF_900095885.1	82.2961
Dickeya dianthicola 16LI01	GCF_018361205.1	92.1643	*Dickeya* sp. CSL RW240	GCF_000406085.2	82.265
Dickeya dianthicola 16JP03	GCF_018361245.1	92.1643	*Dickeya* sp. DW 0440	GCF_000406285.1	82.2098
Dickeya dianthicola 16LI04	GCF_018361165.1	92.1584	*Dickeya lacustris* S29	GCF_003934295.1	81.8755
Dickeya dianthicola DDI_ME30	GCF_018628895.1	92.1583			

Table S2. dDDH values between FZ06 and *D. dadantii* strains.

Query genome	Reference genome	Species name	Isolated source	dDDH
FZ06	GCF_000147055.1	*D. dadantii* 3937	*Saintpaulia ionantha*	69.5
FZ06	GCF_000406145.1	*D. dadantii* NCPPB 898	pelargonium (Comoro Islands)	78.3
FZ06	GCF_000406185.1	*D. dadantii* subsp. *dieffenbachiae* NCPPB 2976^T	dieffenbachia (United States)	73.1
FZ06	GCF_000406265.1	*D. dadantii* NCPPB 3537	potato (Peru)	69.7
FZ06	GCF_001995275.1	*D. dadantii* CZ1501	sweet potato (Zhejiang, China)	70
FZ06	GCF_003049785.1	*D. dadantii* DSM 18020^T	*Pelargonium capitatum* (Comoros)	68.6
FZ06	GCF_009903315.1	*D. dadantii* BI3-1	apple (Japan: Iwate)	69.9
FZ06	GCF_013168455.1	*D. dadantii* Yana2-2	peach (Japan:Fukushima)	69.4
FZ06	GCF_013168465.1	*D. dadantii* Housui2-1	Japanese pear (Japan:Saga)	69.9
FZ06	GCF_013168475.1	*D. dadantii* Kousui1-1	Japanese pear (Japan:Saga)	69.9
FZ06	GCF_013168485.1	*D. dadantii* Aka1-1	peach (Japan:Fukushima)	69.2
FZ06	GCF_013168495.1	*D. dadantii* BI1-1	apple (Japan: Iwate)	69.9
FZ06	GCF_013168575.1	*D. dadantii* Kunimi-3	peach (Japan:Fukushima)	69.7
FZ06	GCA_020406675.1	*D. dadantii* A622-S1-A17	River water (France: Durance River)	86.5
FZ06	GCF_018904205.1	*D. dadantii* S3-1	Calla lily (Taiwan: Taipei)	85.2

Table S3. The functional annotation of the unique genes in FZ06 genome.

Orthologous Groups	Gene	Protein function
OG0003865	FZ06_000005	hypothetical protein
OG0003866	FZ06_000029	hypothetical protein
OG0000277	FZ06_000040, FZ06_003754, FZ06_004455, FZ06_004463	DUF1471 domain-containing protein，
OG0003867	FZ06_000043	RloB domain-containing protein
OG0003868	FZ06_000044	ATP-binding protein
OG0003869	FZ06_000082	hypothetical protein
OG0003870	FZ06_000119	hypothetical protein
OG0003871	FZ06_000121	hypothetical protein
OG0003872	FZ06_000122	hypothetical protein
OG0003873	FZ06_000123	hypothetical protein
OG0003874	FZ06_000124	hypothetical protein
OG0003875	FZ06_000125	hypothetical protein
OG0003876	FZ06_000136	acyltransferase
OG0003877	FZ06_000137	sulfotransferase family 2 domain-containing protein
OG0003878	FZ06_000185	hypothetical protein
OG0003879	FZ06_000221	GNAT family N-acetyltransferase
OG0003880	FZ06_000241	DUF2645 family protein
OG0003881	FZ06_000242	hypothetical protein
OG0003882	FZ06_000249	Rsd/AlgQ family anti-sigma factor
OG0003883	FZ06_000273	cupin domain-containing protein
OG0002407	FZ06_000275, FZ06_000949, FZ06_001656	NAD(P)-dependent alcohol dehydrogenase
OG0000050	FZ06_000330, FZ06_001105, FZ06_002312, FZ06_002867, FZ06_004056	TetR/AcrR family transcriptional regulator
OG0003884	FZ06_000383	helix-turn-helix domain-containing protein
OG0003885	FZ06_000384	hypothetical protein
OG0003886	FZ06_000393	hypothetical protein
OG0003887	FZ06_000398	ParD-like family protein
OG0003888	FZ06_000399	type II toxin-antitoxin system RelE/ParE family toxin
OG0003889	FZ06_000404	hypothetical protein
OG0003890	FZ06_000423	restriction endonuclease subunit S

OG0003891	FZ06_000425	hypothetical protein
OG0000224	FZ06_000426, FZ06_001490, FZ06_001922, FZ06_003011	IS3 family transposase
OG0003892	FZ06_000427	hypothetical protein
OG0003893	FZ06_000429	YjhX family toxin
OG0003894	FZ06_000430	hypothetical protein
OG0003895	FZ06_000432	AAA family ATPase
OG0003896	FZ06_000433	McrC family protein
OG0003897	FZ06_000437	DUF2000 domain-containing protein
OG0003435	FZ06_000442, FZ06_001118	ornithine cyclodeaminase family protein
OG0003436	FZ06_000445	HNH endonuclease
OG0003898	FZ06_000447	DUF4276 family protein
OG0003437	FZ06_000448, FZ06_003800	AAA family ATPase
OG0000341	FZ06_000459, FZ06_000460, FZ06_000461, FZ06_000462	cytolytic delta-endotoxin
OG0004067	FZ06_000472	HNH endonuclease
OG0003429	FZ06_000487, FZ06_004450	DUF2778 domain-containing protein
OG0003899	FZ06_000494	hypothetical protein
OG0003900	FZ06_000511	DUF1328 domain-containing protein
OG0004068	FZ06_000518	flagellar protein FlhE
OG0003901	FZ06_000529	hydroxyisourate hydrolase
OG0003902	FZ06_000532	aromatic ring-hydroxylating dioxygenase subunit alpha
OG0003903	FZ06_000534	FAD-dependent urate hydroxylase HpxO
OG0003904	FZ06_000552	toll/interleukin-1 receptor domain-containing protein
OG0000347	FZ06_000555, FZ06_000556	helix-turn-helix transcriptional regulator
OG0003905	FZ06_000558	S8 family peptidase
OG0002191	FZ06_000559, FZ06_002130	AAA family ATPase
OG0003906	FZ06_000562	hypothetical protein
OG0003907	FZ06_000612	hypothetical protein
OG0002268	FZ06_000661, FZ06_000732, FZ06_004052	FecR domain-containing protein

OG0001456	FZ06_000662, FZ06_000731, FZ06_004053	sigma-70 family RNA polymerase sigma factor
OG0003908	FZ06_000695	hypothetical protein
OG0003909	FZ06_000696	hypothetical protein
OG0003910	FZ06_000702	methyltransferase
OG0003911	FZ06_000703	hypothetical protein
OG0003912	FZ06_000723	IS6 family transposase
OG0003913	FZ06_000724	trypsin-like serine protease
OG0003914	FZ06_000735	addiction module
OG0003915	FZ06_000736	DUF1501 domain-containing protein
OG0003916	FZ06_000737	DUF1800 family protein
OG0003440	FZ06_000750, FZ06_003781	glycoside hydrolase 43 family protein
OG0003441	FZ06_000766, FZ06_000940	DNA gyrase inhibitor SbmC
OG0003917	FZ06_000768	hypothetical protein
OG0003918	FZ06_000769	2Fe-2S iron-sulfur cluster binding
OG0003919	FZ06_000770	hypothetical protein
OG0003920	FZ06_000771	hypothetical protein
OG0003921	FZ06_000787	hypothetical protein
OG0003922	FZ06_000794	type IV toxin-antitoxin system AbiEi family antitoxin domain-containing protein
OG0003923	FZ06_000795	nucleotidyl transferase AbiEii/AbiGii toxin family protein
OG0003924	FZ06_000796	hypothetical protein
OG0000233	FZ06_000797, FZ06_001929, FZ06_003146	toprim domain-containing protein
OG0003488	FZ06_000799, FZ06_001931	host cell division inhibitor Icd-like protein
OG0003925	FZ06_000802	capsid size determination protein
OG0003926	FZ06_000805	tyrosine-type recombinase/integrase
OG0000823	FZ06_000807, FZ06_001621, FZ06_002970	DUF736 domain-containing protein
OG0004069	FZ06_000808	trypsin-like serine protease
OG0003927	FZ06_000809	hypothetical protein
OG0003928	FZ06_000810	DUF2285 domain-containing protein
OG0003929	FZ06_000811	helix-turn-helix domain-containing protein
OG0003930	FZ06_000812	replication initiator protein A
OG0003931	FZ06_000813	S26 family signal peptidase
OG0003932	FZ06_000814	relaxase/mobilization nuclease and DUF3363

OG0003933	FZ06_000816	hypothetical protein
OG0003934	FZ06_000820	entry exclusion lipoprotein TrbK
OG0003935	FZ06_000821	conjugal transfer protein TraG
OG0003936	FZ06_000822	ribbon-helix-helix protein, CopG family
OG0000221	FZ06_000823, FZ06_001596, FZ06_002998	P-type conjugative transfer ATPase TrbB
OG0003937	FZ06_000824	TrbC/VIRB2 family protein
OG0003938	FZ06_000825	VirB3 family type IV secretion system protein
OG0000220	FZ06_000826, FZ06_001603, FZ06_002991	conjugal transfer protein TrbE
OG0003939	FZ06_000827	P-type conjugative transfer protein TrbJ
OG0004070	FZ06_000828	P-type conjugative transfer protein TrbL
OG0003940	FZ06_000829	conjugal transfer protein TrbF
OG0003941	FZ06_000830	P-type conjugative transfer protein TrbG
OG0003942	FZ06_000831	TrbI/VirB10 family protein
OG0003943	FZ06_000832	type II toxin-antitoxin system RelE/ParE family toxin
OG0003944	FZ06_000833	damage-inducible protein J
OG0003945	FZ06_000842	RhsIA family immunity protein
OG0000356	FZ06_000843, FZ06_001618	PAAR domain-containing protein
OG0004071	FZ06_000911	hypothetical protein
OG0003444	FZ06_000937, FZ06_000938	tripartite tricarboxylate transporter substrate binding protein
OG0003463	FZ06_000939, FZ06_001134	MFS transporter
OG0003946	FZ06_000942	hypothetical protein
OG0003947	FZ06_000947	linear amide C-N hydrolase
OG0003948	FZ06_000948	nuclear transport factor 2 family protein
OG0003949	FZ06_000951	Fic family protein
OG0003950	FZ06_000952	zinc chelation protein SecC
OG0003951	FZ06_000953	ash family protein
OG0003952	FZ06_000954	hypothetical protein
OG0003953	FZ06_000958	hypothetical protein
OG0003362	FZ06_000962, FZ06_002666	DUF2786 domain-containing protein
OG0003954	FZ06_000964	hypothetical protein
OG0003955	FZ06_000968	DUF3577 domain-containing protein
OG0003956	FZ06_000969	hypothetical protein
OG0003957	FZ06_000971	DUF29 domain-containing protein
OG0003958	FZ06_000973	type IV pilus biogenesis protein PilM

OG0003959	FZ06_000974	PilN family type IVB pilus formation outer membrane protein
OG0003960	FZ06_000975	type 4b pilus protein PilO2
OG0003961	FZ06_000976	type IV pilus biogenesis protein PilP
OG0003962	FZ06_000978	type II secretion system F family protein
OG0003963	FZ06_000979	pilus assembly protein PilX
OG0003964	FZ06_000980	lytic transglycosylase domain-containing protein
OG0003965	FZ06_000981	prepilin peptidase
OG0003966	FZ06_000987	hypothetical protein
OG0003967	FZ06_000990	hypothetical protein
OG0004072	FZ06_000992	TIGR03758 family integrating conjugative element protein
OG0003968	FZ06_001000	hypothetical protein
OG0003969	FZ06_001001	IS481 family transposase
OG0003970	FZ06_001002	hypothetical protein
OG0003971	FZ06_001003	DUF4935 domain-containing protein
OG0003972	FZ06_001004	hypothetical protein
OG0003973	FZ06_001005	hypothetical protein
OG0003974	FZ06_001007	transcriptional regulator
OG0003975	FZ06_001008	hypothetical protein
OG0003976	FZ06_001009	integrase domain-containing protein
OG0003977	FZ06_001010	hypothetical protein
OG0003978	FZ06_001011	hypothetical protein
OG0003979	FZ06_001012	hypothetical protein
OG0003980	FZ06_001013	hypothetical protein
OG0003981	FZ06_001014	hypothetical protein
OG0003982	FZ06_001016	hypothetical protein
OG0003983	FZ06_001017	hypothetical protein
OG0004073	FZ06_001018	hypothetical protein
OG0003984	FZ06_001019	hypothetical protein
OG0003985	FZ06_001020	hypothetical protein
OG0003986	FZ06_001022	hypothetical protein
OG0003987	FZ06_001023	hypothetical protein
OG0003988	FZ06_001099	hypothetical protein
OG0003989	FZ06_001104	nitroreductase family protein
OG0003462	FZ06_001122, FZ06_001124	isocyanide synthase family protein
OG0003990	FZ06_001139	alpha/beta fold hydrolase
OG0003991	FZ06_001179	nuclear transport factor 2 family protein
OG0003992	FZ06_001181	metallohydrolase
OG0003993	FZ06_001233	hypothetical protein
OG0003465	FZ06_001264	spermidine N1-acetyltransferase
OG0003994	FZ06_001293	hypothetical protein

OG0003995	FZ06_001356	endonuclease VIII
OG0003996	FZ06_001385	CDF family zinc transporter ZitB
OG0004074	FZ06_001403	S8/S53 family peptidase
OG0003997	FZ06_001442	hypothetical protein
OG0003998	FZ06_001449	hemin-degrading factor
OG0003999	FZ06_001459	hypothetical protein
OG0003466	FZ06_001474, FZ06_001481	hypothetical protein
OG0003467	FZ06_001477, FZ06_001484	toprim domain-containing protein
OG0003468	FZ06_001478, FZ06_001485	site-specific tyrosine recombinase XerC
OG0003469	FZ06_001479, FZ06_001486	hypothetical protein
OG0004000	FZ06_001480	hypothetical protein
OG0004075	FZ06_001487	hypothetical protein
OG0004001	FZ06_001488	SMI1/KNR4 family protein
OG0003470	FZ06_001509	hypothetical protein
OG0003499	FZ06_001510	TetR family transcriptional regulator
OG0004002	FZ06_001523	hypothetical protein
OG0004003	FZ06_001524	hypothetical protein
OG0004004	FZ06_001536	ABC transporter permease
OG0004005	FZ06_001537	alpha/beta fold hydrolase
OG0004006	FZ06_001561	hypothetical protein
OG0004007	FZ06_001583	DUF4297 domain-containing protein
OG0004008	FZ06_001584	hypothetical protein
OG0004009	FZ06_001585	hypothetical protein
OG0003471	FZ06_001587	hypothetical protein
OG0000831	FZ06_001588, FZ06_003004	DUF1738 domain-containing protein
OG0003472	FZ06_001591	RNA-directed DNA polymerase
OG0002779	FZ06_001593, FZ06_003000	type IV secretory system conjugative DNA transfer family protein
OG0004010	FZ06_001594	hypothetical protein
OG0000830	FZ06_001595, FZ06_002999	conjugal transfer protein
OG0000829	FZ06_001597, FZ06_002997	TrbI/VirB10 family protein
OG0000828	FZ06_001598, FZ06_002996	P-type conjugative transfer protein VirB9
OG0000827	FZ06_001599, FZ06_002995	type IV secretion system protein
OG0000826	FZ06_001600,	type IV secretion system protein

	FZ06_002994	
OG0000825	FZ06_001601, FZ06_002993	EexN family lipoprotein
OG0000824	FZ06_001602, FZ06_002992	type IV secretion system protein
OG0004076	FZ06_001607	SIR2 family protein
OG0004011	FZ06_001619	SMI1/KNR4 family protein
OG0004012	FZ06_001647	hypothetical protein
OG0004013	FZ06_001648	FAD-dependent monooxygenase
OG0004014	FZ06_001650	YdcF family protein
OG0004077	FZ06_001664	ABC transporter substrate-binding protein
OG0003478	FZ06_001675	DUF3916 domain-containing protein
OG0004015	FZ06_001676	hypothetical protein
OG0004016	FZ06_001677	hypothetical protein
OG0004017	FZ06_001692	hypothetical protein
OG0004018	FZ06_001714	permease
OG0004019	FZ06_001715	FAD/NAD(P)-binding protein
OG0004020	FZ06_001724	EVE domain-containing protein
OG0003481	FZ06_001726, FZ06_001747	ABC transporter permease
OG0003482	FZ06_001729, FZ06_004051	energy transducer TonB
OG0003483	FZ06_001730, FZ06_001748	ABC transporter substrate-binding protein
OG0004021	FZ06_001732	Tat pathway signal sequence domain protein
OG0004022	FZ06_001733	hypothetical protein
OG0004078	FZ06_001744	DUF2167 domain-containing protein
OG0004023	FZ06_001745	aryl-sulfate sulfotransferase
OG0004079	FZ06_001751	amidohydrolase
OG0003484	FZ06_001776, FZ06_001777	metal ABC transporter permease
OG0004024	FZ06_001785	MFS transporter
OG0004025	FZ06_001796	hypothetical protein
OG0004026	FZ06_001799	YqaE/Pmp3 family membrane protein
OG0004027	FZ06_001807	PD-(D/E)XK nuclease family protein
OG0003485	FZ06_001821	glyoxalase
OG0004028	FZ06_001850	helix-turn-helix domain-containing protein
OG0004029	FZ06_001851	DMT family transporter
OG0004030	FZ06_001857	Eco57I restriction-modification methylase domain-containing protein
OG0004031	FZ06_001858	PaeR7I family type II restriction endonuclease
OG0004032	FZ06_001864	hypothetical protein
OG0004033	FZ06_001873	hypothetical protein

OG0002921	FZ06_001892, FZ06_001893, FZ06_001894	hypothetical protein
OG0004034	FZ06_001907	hypothetical protein
OG0003486	FZ06_001909	protease inhibitor I42 family protein
OG0003487	FZ06_001910	hypothetical protein
OG0004035	FZ06_001911	hypothetical protein
OG0000009	FZ06_001914, FZ06_002299	IS3 family transposase
OG0004036	FZ06_001915	gamma-glutamylcyclotransferase
OG0004037	FZ06_001924	hypothetical protein
OG0004038	FZ06_001925	type II toxin-antitoxin system RelE/ParE family toxin
OG0004039	FZ06_001927	HD domain-containing protein
OG0004040	FZ06_001928	nucleotidyltransferase domain-containing protein
OG0003489	FZ06_001932	AlpA family phage regulatory protein
OG0004041	FZ06_001933	hypothetical protein
OG0004042	FZ06_001953	phosphatase PAP2 family protein
OG0004043	FZ06_001967	hypothetical protein
OG0004044	FZ06_001968	3-oxoacyl-ACP synthase
OG0003490	FZ06_001969	HAD-IIIC family phosphatase
OG0004045	FZ06_001970	acyl carrier protein
OG0004046	FZ06_001972	alpha/beta hydrolase
OG0004047	FZ06_002050	amidohydrolase
OG0004080	FZ06_002060	his operon leader peptide
OG0004048	FZ06_002092	cyanophycin synthetase
OG0004049	FZ06_002104	bacteriophage abortive infection AbiH family protein
OG0004050	FZ06_002116	hypothetical protein
OG0004051	FZ06_002120	hypothetical protein
OG0004052	FZ06_002128	hypothetical protein
OG0004053	FZ06_002129	hypothetical protein
OG0004054	FZ06_002131	hypothetical protein
OG0004055	FZ06_002132	DUF4157 domain-containing protein
OG0004056	FZ06_002165	DNA/RNA non-specific endonuclease
OG0004057	FZ06_002169	FIST C-terminal domain-containing protein
OG0004058	FZ06_002171	aminopeptidase
OG0004059	FZ06_002173	hypothetical protein
OG0004060	FZ06_002174	GlsB/YeaQ/YmgE family stress response membrane protein
OG0004061	FZ06_002205	hypothetical protein
OG0004062	FZ06_002222	hypothetical protein

OG0004063	FZ06_002228	anthranilate phosphoribosyltransferase
OG0004064	FZ06_002237	hypothetical protein
OG0004065	FZ06_002276	ATP-binding protein
OG0003492	FZ06_002279, FZ06_002287	hypothetical protein
OG0003500	FZ06_002280, FZ06_002289	Replication-associated protein G2P
OG0003351	FZ06_002282, FZ06_002292	hypothetical protein
OG0004066	FZ06_002283	DUF2523 domain-containing protein
OG0003352	FZ06_002284, FZ06_002294	hypothetical protein
OG0003353	FZ06_002286, FZ06_002296	hypothetical protein
OG0003493	FZ06_002288	hypothetical protein
OG0003741	FZ06_002290	DNA-binding protein
OG0003742	FZ06_002291	hypothetical protein
OG0003743	FZ06_002301	hypothetical protein
OG0003744	FZ06_002305	ClbS/DfsB family four-helix bundle protein
OG0003745	FZ06_002307	DUF3313 domain-containing protein
OG0003746	FZ06_002311	membrane integrity-associated transporter subunit PqiC
OG0003747	FZ06_002317	NAD(P)H-dependent oxidoreductase
OG0003748	FZ06_002390	hypothetical protein
OG0003355	FZ06_002391, FZ06_002472	hypothetical protein
OG0004081	FZ06_002398	hypothetical protein
OG0004082	FZ06_002447	pheST operon leader peptide PheM
OG0004083	FZ06_002475	cupin domain-containing protein
OG0003749	FZ06_002505	hypothetical protein
OG0003750	FZ06_002522	hypothetical protein
OG0003357	FZ06_002533	oxidoreductase
OG0003751	FZ06_002535	hypothetical protein
OG0003752	FZ06_002536	hypothetical protein
OG0003753	FZ06_002538	type II toxin-antitoxin system Phd/YefM family antitoxin
OG0003754	FZ06_002569	hypothetical protein
OG0003358	FZ06_002575	esterase-like activity of phytase family protein
OG0003359	FZ06_002609	acid resistance repetitive basic protein Asr
OG0004084	FZ06_002663	aconitate hydratase AcnA
OG0003360	FZ06_002664	DNA-binding protein
OG0003361	FZ06_002665	regulatory protein GemA
OG0003363	FZ06_002667	hypothetical protein

OG0003364	FZ06_002668	host-nuclease inhibitor Gam family protein
OG0003365	FZ06_002669	hypothetical protein
OG0003366	FZ06_002670	hypothetical protein
OG0003367	FZ06_002671	AAA family ATPase
OG0003368	FZ06_002672	DDE-type integrase/transposase/recombinase
OG0003755	FZ06_002673	DUF3102 domain-containing protein
OG0003369	FZ06_002674	helix-turn-helix domain-containing protein
OG0003370	FZ06_002675	DNA-binding protein
OG0003756	FZ06_002676	helix-turn-helix transcriptional regulator
OG0003371	FZ06_002677	hypothetical protein
OG0003372	FZ06_002678	hypothetical protein
OG0003373	FZ06_002679	putative holin
OG0003374	FZ06_002680	transglycosylase SLT domain-containing protein
OG0003502	FZ06_002681	hypothetical protein
OG0003375	FZ06_002682	hypothetical protein
OG0003376	FZ06_002683	hypothetical protein
OG0003377	FZ06_002684	DUF3486 family protein
OG0003378	FZ06_002685	hypothetical protein
OG0003379	FZ06_002686	DUF935 domain-containing protein
OG0003380	FZ06_002687	minor capsid protein
OG0003381	FZ06_002688	phage virion morphogenesis protein
OG0003382	FZ06_002689	peptidase
OG0003383	FZ06_002690	hypothetical protein
OG0003384	FZ06_002691	DUF2190 family protein
OG0003385	FZ06_002692	DUF1320 domain-containing protein
OG0003386	FZ06_002693	Gp37 family protein
OG0003387	FZ06_002694	hypothetical protein
OG0003388	FZ06_002695	phage tail sheath subtilisin-like domain-containing protein
OG0003389	FZ06_002696	phage major tail tube protein
OG0003757	FZ06_002697	hypothetical protein
OG0003390	FZ06_002698	phage tail assembly protein
OG0004085	FZ06_002699	GpE family phage tail protein
OG0003391	FZ06_002700	hypothetical protein
OG0003392	FZ06_002701	phage tail tape measure protein
OG0003393	FZ06_002702	phage tail protein
OG0003394	FZ06_002703	tail protein X
OG0003395	FZ06_002704	phage late control D family protein
OG0003396	FZ06_002705	phage baseplate assembly protein V
OG0003758	FZ06_002706	hypothetical protein
OG0003397	FZ06_002707	GPW/gp25 family protein
OG0003398	FZ06_002708	baseplate J/gp47 family protein
OG0003399	FZ06_002709	phage tail protein

OG0003759	FZ06_002710	hypothetical protein
OG0003760	FZ06_002725	YnhF family membrane protein
OG0003761	FZ06_002732	B3/4 domain-containing protein
OG0004086	FZ06_002735	type II secretion system pilot lipoprotein GspS
OG0003762	FZ06_002736	hypothetical protein
OG0003763	FZ06_002737	type II secretion system protein M
OG0003764	FZ06_002738	general secretion pathway protein GspL
OG0003765	FZ06_002746	hypothetical protein
OG0003400	FZ06_002748,<br>FZ06_003220	carbohydrate porin
OG0003401	FZ06_002757,<br>FZ06_002759	Fe-S cluster assembly protein SufD
OG0003766	FZ06_002784	hypothetical protein
OG0003767	FZ06_002789	hypothetical protein
OG0000714	FZ06_002810,<br>FZ06_002811,<br>FZ06_002812	glycosyl transferase
OG0003768	FZ06_002871	FkbM family methyltransferase
OG0003402	FZ06_002876	Gfo/Idh/MocA family oxidoreductase
OG0003403	FZ06_002877	ATP-grasp domain-containing protein
OG0003769	FZ06_002930	hypothetical protein
OG0003770	FZ06_002938	hypothetical protein
OG0003771	FZ06_002972	hypothetical protein
OG0003772	FZ06_002984	DUF2971 domain-containing protein
OG0003773	FZ06_002985	hypothetical protein
OG0003774	FZ06_002986	nucleotidyltransferase domain-containing protein
OG0003775	FZ06_002987	pentapeptide repeat-containing protein
OG0003776	FZ06_003005	hypothetical protein
OG0003777	FZ06_003007	hypothetical protein
OG0003778	FZ06_003008	phosphoadenosine phosphosulfate reductase family protein
OG0003779	FZ06_003009	ATP-binding protein
OG0003780	FZ06_003010	DUF4007 family protein
OG0003781	FZ06_003041	type II toxin-antitoxin system HipA family toxin
OG0003782	FZ06_003042	helix-turn-helix transcriptional regulator
OG0003783	FZ06_003061	hypothetical protein
OG0003784	FZ06_003079	hypothetical protein
OG0003785	FZ06_003132	malate dehydrogenase (quinone)
OG0003786	FZ06_003136	hypothetical protein
OG0003404	FZ06_003137	hypothetical protein
OG0003405	FZ06_003139	hypothetical protein
OG0003406	FZ06_003140	antA/AntB antirepressor family protein
OG0003787	FZ06_003141	hypothetical protein

OG0003788	FZ06_003142	host cell division inhibitor Icd-like protein
OG0003407	FZ06_003144	hypothetical protein
OG0003408	FZ06_003145	hypothetical protein
OG0003789	FZ06_003147	single-stranded DNA-binding protein
OG0004087	FZ06_003148	helix-turn-helix domain-containing protein
OG0004088	FZ06_003149	peptidase M41 family protein
OG0004089	FZ06_003150	hypothetical protein
OG0003790	FZ06_003151	hypothetical protein
OG0003409	FZ06_003152	WP_013317580.1
OG0003503	FZ06_003153	DUF4224 domain-containing protein
OG0003791	FZ06_003155	hypothetical protein
OG0003792	FZ06_003165	outer membrane porin, OprD family
OG0004099	FZ06_003191	hypothetical protein
OG0003793	FZ06_003199	hypothetical protein
OG0003794	FZ06_003200	methyltransferase domain-containing protein
OG0003795	FZ06_003202	PIN domain-containing protein
OG0003796	FZ06_003214	Glu/Leu/Phe/Val dehydrogenase
OG0003797	FZ06_003222	beta-galactosidase
OG0003798	FZ06_003223	glycosyl hydrolase 53 family protein
OG0003799	FZ06_003226	extracellular solute-binding protein
OG0003800	FZ06_003232	GNAT family N-acetyltransferase
OG0003801	FZ06_003240	ATP-dependent Clp protease proteolytic subunit
OG0003411	FZ06_003267, FZ06_003268	YolA family protein
OG0003412	FZ06_003292, FZ06_003293	spore coat protein U domain-containing protein
OG0003802	FZ06_003294	molecular chaperone
OG0003803	FZ06_003295	fimbrial biogenesis outer membrane usher protein
OG0003804	FZ06_003296	spore coat U domain-containing protein
OG0003805	FZ06_003308	hypothetical protein
OG0003413	FZ06_003312	pyridoxal phosphate-dependent aminotransferase
OG0001000	FZ06_003313, FZ06_004273	multidrug effflux MFS transporter
OG0003414	FZ06_003314	Gfo/Idh/MocA family oxidoreductase
OG0003415	FZ06_003315	hypothetical protein
OG0003416	FZ06_003316	class I SAM-dependent methyltransferase
OG0004090	FZ06_003321	MFS transporter
OG0003806	FZ06_003323	hypothetical protein
OG0003807	FZ06_003325	bifunctional helix-turn-helix transcriptional regulator/GNAT family N-acetyltransferase
OG0003808	FZ06_003329	acyltransferase
OG0004091	FZ06_003379	acid phosphatase AphA
OG0003809	FZ06_003424	Fe(3+)-hydroxamate ABC transporter

		substrate-binding protein FhuD
OG0003810	FZ06_003448	glutaredoxin-like protein NrdH
OG0004092	FZ06_003449	class Ib ribonucleoside-diphosphate reductase assembly flavoprotein NrdI
OG0003811	FZ06_003490	hypothetical protein
OG0003812	FZ06_003494	GNAT family N-acetyltransferase
OG0004093	FZ06_003500	winged helix-turn-helix transcriptional regulator
OG0003417	FZ06_003513	hypothetical protein
OG0003813	FZ06_003530	GIY-YIG nuclease family protein
OG0003814	FZ06_003537	hypothetical protein
OG0003815	FZ06_003538	hypothetical protein
OG0003816	FZ06_003539	hypothetical protein
OG0003817	FZ06_003540	DUF2157 domain-containing protein
OG0003818	FZ06_003545	MobA/MobL family protein
OG0003819	FZ06_003546	DUF262 domain-containing protein
OG0003820	FZ06_003547	hypothetical protein
OG0003821	FZ06_003549	hypothetical protein
OG0004094	FZ06_003552	hypothetical protein
OG0003822	FZ06_003554	hypothetical protein
OG0003823	FZ06_003562	YfiR family protein
OG0004095	FZ06_003609	YfiM family lipoprotein
OG0003824	FZ06_003665	FlxA-like family protein
OG0003825	FZ06_003712	Fic family protein
OG0004100	FZ06_003713	hypothetical protein
OG0003826	FZ06_003746	RES family NAD+ phosphorylase
OG0003827	FZ06_003747	DUF2384 domain-containing protein
OG0003828	FZ06_003775	PTS sugar transporter subunit IIA
OG0003829	FZ06_003776	glycoside hydrolase family 88 protein
OG0003830	FZ06_003780	DUF4962 domain-containing protein
OG0003831	FZ06_003784	hypothetical protein
OG0003832	FZ06_003801	DUF4276 family protein
OG0003833	FZ06_003830	alpha/beta hydrolase
OG0003834	FZ06_003913	acyltransferase
OG0003835	FZ06_003965	hypothetical protein
OG0001440	FZ06_003992	GDP-mannose 4,6-dehydratase
OG0003836	FZ06_003995	GDP-L-fucose synthase
OG0003837	FZ06_003996	hypothetical protein
OG0003838	FZ06_004000	hypothetical protein
OG0003839	FZ06_004017	nitrogen fixation protein NifQ
OG0003424	FZ06_004018, FZ06_004033	nitrogenase cofactor biosynthesis protein NifB
OG0003840	FZ06_004025	nitrogen fixation protein NifZ
OG0003841	FZ06_004026	nitrogen fixation protein NifW

OG0003425	FZ06_004034, FZ06_004042	nitrogenase iron-molybdenum cofactor biosynthesis protein NifN ， nitrogenase molybdenum-iron protein subunit beta
OG0003426	FZ06_004035, FZ06_004043	nitrogenase iron-molybdenum cofactor biosynthesis protein NifE ， nitrogenase molybdenum-iron protein alpha chain
OG0003842	FZ06_004038	type II toxin-antitoxin system HipA family toxin
OG0003843	FZ06_004040	nitrogen fixation protein NifY
OG0003844	FZ06_004041	NifT/FixU family protein
OG0003845	FZ06_004044	nitrogenase iron protein
OG0003846	FZ06_004057	NAD(P)H-dependent oxidoreductase
OG0003847	FZ06_004058	saccharopine dehydrogenase
OG0004096	FZ06_004075	beta-mannosidase
OG0003848	FZ06_004152	CPBP family intramembrane metalloprotease
OG0003849	FZ06_004216	EAL domain-containing protein
OG0003850	FZ06_004218	hypothetical protein
OG0003851	FZ06_004234	hypothetical protein
OG0003852	FZ06_004244	HPP family protein
OG0003853	FZ06_004272	transporter
OG0003854	FZ06_004297	hypothetical protein
OG0003855	FZ06_004395	hypothetical protein
OG0003856	FZ06_004447	hypothetical protein
OG0001745	FZ06_004449	Hcp family type VI secretion system effector
OG0003857	FZ06_004467	zinc ribbon domain-containing protein
OG0004097	FZ06_004468	bile acid:sodium symporter
OG0003858	FZ06_004469	CehA/McbA family metallohydrolase
OG0003859	FZ06_004537	NAD(P)H-dependent oxidoreductase
OG0003860	FZ06_004538	pyridoxal-phosphate dependent enzyme
OG0003431	FZ06_004540, FZ06_004543	ATP-grasp domain-containing protein
OG0003861	FZ06_004541	SUMF1/EgtB/PvdO family nonheme iron enzyme
OG0003862	FZ06_004542	hypothetical protein
OG0003863	FZ06_004544	MFS transporter
OG0004098	FZ06_004576	helix-turn-helix domain-containing protein
OG0003864	FZ06_004577	type II toxin-antitoxin system HipA family toxin

Table S4. GO functional annotation of specific genes within strain FZ06.

Class	GO ID	Counts	Gene
Biological process	GO:0065007	9	FZ06_000249; FZ06_000562; FZ06_000766; FZ06_000822; FZ06_000833; FZ06_000940; FZ06_001348; FZ06_002674; FZ06_003448.
Cellular process	GO:0009987	56	FZ06_000051; FZ06_000249; FZ06_000426; FZ06_000529; FZ06_000562; FZ06_000662; FZ06_000731; FZ06_000805; FZ06_000809; FZ06_000813; FZ06_000822; FZ06_000823; FZ06_000828; FZ06_000833; FZ06_000900; FZ06_000974; FZ06_001001; FZ06_001009; FZ06_001356; FZ06_001478; FZ06_001485; FZ06_001490; FZ06_001536; FZ06_001596; FZ06_001600; FZ06_001729; FZ06_001730; FZ06_001748; FZ06_001922; FZ06_001953; FZ06_001968; FZ06_002228; FZ06_002533; FZ06_002663; FZ06_002668; FZ06_002672; FZ06_002674; FZ06_002737; FZ06_002748; FZ06_002757; FZ06_002759; FZ06_002994; FZ06_002998; FZ06_003011; FZ06_003132; FZ06_003214; FZ06_003238; FZ06_003448; FZ06_003449; FZ06_003875; FZ06_003992; FZ06_004034; FZ06_004035; FZ06_004064; FZ06_004152; FZ06_004462
Localization	GO:0051179	11	FZ06_000828; FZ06_001449; FZ06_001536; FZ06_001596; FZ06_001600; FZ06_001729; FZ06_002737; FZ06_002748; FZ06_002994; FZ06_002998; FZ06_003220
Metabolic process	GO:0008152	64	FZ06_000051; FZ06_000249; FZ06_000426; FZ06_000529; FZ06_000532; FZ06_000562; FZ06_000662; FZ06_000702; FZ06_000731; FZ06_000735; FZ06_000750; FZ06_000805; FZ06_000813; FZ06_000822; FZ06_000833; FZ06_000900; FZ06_001001; FZ06_001009; FZ06_001179; FZ06_001356; FZ06_001478; FZ06_001485; FZ06_001490; FZ06_001730; FZ06_001748; FZ06_001922; FZ06_001953; FZ06_001968; FZ06_002228; FZ06_002533; FZ06_002663; FZ06_002668; FZ06_002672; FZ06_002674; FZ06_002757; FZ06_002759; FZ06_003011; FZ06_003132; FZ06_003214; FZ06_003222; FZ06_003223; FZ06_003238; FZ06_003316; FZ06_003449; FZ06_003781; FZ06_003875; FZ06_003992; FZ06_004017;

			FZ06_004018; FZ06_004025; FZ06_004026; FZ06_004033; FZ06_004034; FZ06_004035; FZ06_004040; FZ06_004041; FZ06_004042; FZ06_004043; FZ06_004044; FZ06_004064; FZ06_004075; FZ06_004152; FZ06_004462; FZ06_004544; FZ06_000051; FZ06_000249; FZ06_000426; FZ06_000529; FZ06_000532; FZ06_000562; FZ06_000662; FZ06_000702; FZ06_000731; FZ06_000735; FZ06_000750; FZ06_000805; FZ06_000813; FZ06_000822; FZ06_000833; FZ06_000900; FZ06_001001; FZ06_001009; FZ06_001179; FZ06_001356; FZ06_001478; FZ06_001485; FZ06_001490; FZ06_001730; FZ06_001748; FZ06_001922; FZ06_001953; FZ06_001968; FZ06_002228; FZ06_002533; FZ06_002663; FZ06_002668; FZ06_002672; FZ06_002674; FZ06_002757; FZ06_002759; FZ06_003011; FZ06_003132; FZ06_003214; FZ06_003222; FZ06_003223; FZ06_003238; FZ06_003316; FZ06_003449; FZ06_003781; FZ06_003875; FZ06_003992; FZ06_004017; FZ06_004018; FZ06_004025; FZ06_004026; FZ06_004033; FZ06_004034; FZ06_004035; FZ06_004040; FZ06_004041; FZ06_004042; FZ06_004043; FZ06_004044; FZ06_004064; FZ06_004075; FZ06_004152; FZ06_004462; FZ06_004544
Response to stimulus	GO:0050896	4	FZ06_000662; FZ06_000731; FZ06_001356; FZ06_002668
Cellular anatomical entity	GO:0110165	85	FZ06_000124; FZ06_000136; FZ06_000137; FZ06_000241; FZ06_000249; FZ06_000459; FZ06_000460; FZ06_000461; FZ06_000462; FZ06_000511; FZ06_000612; FZ06_000735; FZ06_000766; FZ06_000821; FZ06_000823; FZ06_000824; FZ06_000828; FZ06_000829; FZ06_000831; FZ06_000939; FZ06_000940; FZ06_000969; FZ06_000974; FZ06_000975; FZ06_000978; FZ06_000979; FZ06_000981; FZ06_000987; FZ06_000992; FZ06_001011; FZ06_001012; FZ06_001014; FZ06_001023; FZ06_001104; FZ06_001134; FZ06_001348; FZ06_001385; FZ06_001509; FZ06_001523; FZ06_001524; FZ06_001536; FZ06_001537; FZ06_001593; FZ06_001597; FZ06_001599;

			FZ06_001600; FZ06_001603; FZ06_001714; FZ06_001729; FZ06_001744; FZ06_001799; FZ06_001851; FZ06_001953; FZ06_002174; FZ06_002205; FZ06_002398; FZ06_002677; FZ06_002679; FZ06_002682; FZ06_002700; FZ06_002701; FZ06_002725; FZ06_002736; FZ06_002737; FZ06_002738; FZ06_002746; FZ06_002748; FZ06_002991; FZ06_002994; FZ06_002995; FZ06_002997; FZ06_003000; FZ06_003146; FZ06_003165; FZ06_003178; FZ06_003220; FZ06_003321; FZ06_003329; FZ06_003379; FZ06_003913; FZ06_004152; FZ06_004244; FZ06_004467; FZ06_004468; FZ06_004544
Protein-containing complex	GO:0032991	10	FZ06_001536; FZ06_001596; FZ06_002737; FZ06_002748; FZ06_002998; FZ06_003146; FZ06_003220; FZ06_003222; FZ06_004042; FZ06_004043
ATP-dependent activity	GO:0140657	9	FZ06_000044; FZ06_000432; FZ06_000448; FZ06_000797; FZ06_001348; FZ06_001509; FZ06_003146; FZ06_003240; FZ06_003800
Binding	GO:0005488	82	FZ06_000044; FZ06_000051; FZ06_000383; FZ06_000426; FZ06_000427; FZ06_000432; FZ06_000448; FZ06_000472; FZ06_000532; FZ06_000534; FZ06_000555; FZ06_000556; FZ06_000557; FZ06_000621; FZ06_000662; FZ06_000731; FZ06_000769; FZ06_000786; FZ06_000797; FZ06_000805; FZ06_000818; FZ06_000826; FZ06_000833; FZ06_000839; FZ06_000868; FZ06_000900; FZ06_000951; FZ06_000955; FZ06_001001; FZ06_001009; FZ06_001356; FZ06_001403; FZ06_001478; FZ06_001485; FZ06_001490; FZ06_001509; FZ06_001510; FZ06_001591; FZ06_001596; FZ06_001603; FZ06_001614; FZ06_001850; FZ06_001922; FZ06_001969; FZ06_002092; FZ06_002165; FZ06_002228; FZ06_002663; FZ06_002664; FZ06_002668; FZ06_002672; FZ06_002674; FZ06_002675; FZ06_002676; FZ06_002732; FZ06_002967; FZ06_002978; FZ06_002991; FZ06_002998; FZ06_003009; FZ06_003011; FZ06_003042; FZ06_003146; FZ06_003147; FZ06_003202; FZ06_003214; FZ06_003222; FZ06_003238; FZ06_003379;

			FZ06_003449; FZ06_003800; FZ06_003992; FZ06_004017; FZ06_004018; FZ06_004042; FZ06_004043; FZ06_004044; FZ06_004064; FZ06_004462; FZ06_004540; FZ06_004543; FZ06_004576
Catalytic activity	GO:0003824	89	FZ06_000044; FZ06_000051; FZ06_000136; FZ06_000137; FZ06_000221; FZ06_000426; FZ06_000432; FZ06_000445; FZ06_000448; FZ06_000472; FZ06_000529; FZ06_000532; FZ06_000534; FZ06_000702; FZ06_000735; FZ06_000750; FZ06_000769; FZ06_000797; FZ06_000808; FZ06_000813; FZ06_000900; FZ06_000947; FZ06_000981; FZ06_001012; FZ06_001104; FZ06_001124; FZ06_001181; FZ06_001356; FZ06_001403; FZ06_001477; FZ06_001484; FZ06_001490; FZ06_001537; FZ06_001591; FZ06_001745; FZ06_001751; FZ06_001922; FZ06_001953; FZ06_001968; FZ06_001969; FZ06_002050; FZ06_002092; FZ06_002165; FZ06_002228; FZ06_002533; FZ06_002569; FZ06_002663; FZ06_002732; FZ06_003008; FZ06_003011; FZ06_003041; FZ06_003132; FZ06_003146; FZ06_003202; FZ06_003214; FZ06_003222; FZ06_003223; FZ06_003232; FZ06_003238; FZ06_003240; FZ06_003316; FZ06_003325; FZ06_003329; FZ06_003448; FZ06_003494; FZ06_003781; FZ06_003800; FZ06_003913; FZ06_003992; FZ06_003995; FZ06_004018; FZ06_004034; FZ06_004035; FZ06_004038; FZ06_004042; FZ06_004043; FZ06_004044; FZ06_004064; FZ06_004075; FZ06_004152; FZ06_004462; FZ06_004469; FZ06_004537; FZ06_004538; FZ06_004540; FZ06_004541; FZ06_004543; FZ06_004544; FZ06_004577
Molecular function regulator	GO:0098772	3	FZ06_000766; FZ06_000809; FZ06_000940
Toxin activity	GO:0090729	1	FZ06_003202
Transcription regulator activity	GO:0140110	6	FZ06_000185; FZ06_000662; FZ06_000731; FZ06_001850; FZ06_003325; FZ06_003500
Transporter activity	GO:0005215	10	FZ06_000939; FZ06_001134; FZ06_001348; FZ06_001385; FZ06_001509; FZ06_002398; FZ06_002748; FZ06_003220; FZ06_004468; FZ06_004544

Table S5. The homologs of cell wall degrading enzymes in the genomes of FZ06 and MS2.

Gene	Accesion no.		Identity (%)	Function
	FZ06	MS2		
pnl	FZ06-000597	C1O30_RS02835	96	pectate lyase
pelN	FZ06-002084	C1O30_RS09885	92	pectate lyase
pelW	FZ06-002431	C1O30_RS11330	90	pectate lyase
pelL	FZ06-002934	C1O30_RS13585	95	pectate lyase
pelI	FZ06-003230	C1O30_RS14580	84	pectate lyase
pelA	FZ06-003367	C1O30_RS15345	92	pectate lyase
pelE	FZ06-003368	C1O30_RS15350	94	pectate lyase
pelD	FZ06-003369	C1O30_RS15355	79	pectate lyase
paeX	FZ06-002437	C1O30-RS11360	88	pectin acetylesterase
paeY	FZ06-003370	C1O30_RS15360	82	pectin acetylesterase
pemA	FZ06-003371	C1O30_RS15365	94	pectinesterase
pelC	FZ06-004176	C1O30_RS19205	95	pectate lyase
pelB	FZ06-004177	C1O30_RS19210	97	pectate lyase
pelZ	FZ06-004178	C1O30_RS19215	95	pectate lyase
pelX	FZ06-004533	C1O30_RS21105	92	pectate lyase
pehN	FZ06-001235	C1O30_RS05710	97	polygalacturonase
pehK	FZ06-003642	C1O30_RS16710	87	polygalacturonase
pehV	FZ06-004295	/	68	polygalacturonase
pehW	FZ06-004296	/	79	polygalacturonase
pehX	FZ06-004298	C1O30_RS19785	88	polygalacturonase
prtX	FZ06-002318	C1O30_RS10785	91	serine 3-dehydrogenase
prtC	FZ06-002319	C1O30_RS10790	92	serine 3-dehydrogenase
prtB	FZ06-002320	C1O30_RS10795	91	serine 3-dehydrogenase
prtG	FZ06-002325	C1O30_RS10820	90	serine 3-dehydrogenase
bglA	FZ06-000406	C1O30_RS02015	95	6-phospho-beta-glucosidase
bgxA	FZ06-001810	C1O30_RS08765	91	beta-glucosidase
bglB	FZ06-002749	C1O30_RS12705	94	6-phospho-beta-glucosidase
lfaA	FZ06-002816	C1O30_RS12980	93	alpha-glucosidase
nagZ	FZ06-002822	C1O30_RS13010	94	beta-hexosaminidase
celZ	FZ06-002933	C1O30_RS13580	82	endoglucanase
bglC	FZ06-003725	/	54	6-phospho-beta-glucosidase
bglD	FZ06-003726	/	53	6-phospho-beta-glucosidase
celH	FZ06-003734	C1O30_RS17155	95	beta-glucosidase
celY	FZ06-004412	C1O30_RS20545	88	endoglucanase
rhiE1	FZ06-000918	C1O30_ RS04480	90	rhamnogalacturonate lyase
rhiE2	/	C1O30_ RS10600	51	rhamnogalacturonate lyase
xynA	FZ06-001782	C1O30_ RS11050	83	xylanase

Table S6. Primers used in this study.

Mutant	Primer	Primer sequence (5'-3')	Gene segment
Reference gene	16S-F	GGTGAAATGCGTAGAGAT	16S
	16S-R	TGTCAAGAGTAGGTAAGGT	
RT-PCR primers for detection of CWDE genes	pnl-F	CGCTGGGGGCGAATAAATC	*pnl*
	pnl-R	GTGAGGTGATCCAGCCAGTATT	
	pelN-F	AGCCAGACGGATATCGTGAA	*pelN*
	pelN-R	AAATCCTTTAACGCGGCAATC	
	pelL-F	TAGCCAGCGAGGTATCC	*pelL*
	pelL-R	CGTCATATAAAAACGGCATT	
	pelI-F	AAACCTGCGTATTTCCGCCT	*pelI*
	pelI-R	TGGCGTTATGCGCGATCC	
	pelA-F	TGACATGTACACCACCAAAGA	*pelA*
	pelA-R	GACCAATCAGCATGGTTTTGT	
	pelE -F	GACCATGTCACCATCAGCGA	*pelE*
	pelE -R	GTTGTTGTGGAAGGTGACGC	
	pelD -F	GATCGGCCACAGCGACAG	*pelD*
	pelD -R	CGTCGCCCAGATACACGTT	
	pelC -F	GCCAATATCTGCGGCCAGT	*pelC*
	pelC -R	AGCCGATACGCATGTTTTGC	
	pelB -F	ACTTCACCAAAGGCATCACCA	*pelB*
	pelB -R	TTCGGCGAGTTGTCGATACG	
	pelZ -F	CGACTCCAGCCACTCCAAAG	*pelZ*
	pelZ -R	CCTGGGTTGTAGATCAGGTTGT	
	pelW -F	TGGCAAATGGCGGAAATTTTA	*pelW*
	pelW -R	AATAACCCGGCTTTCTCCAT	
	pelX -F	GATGCCGCCGGAAACTCTT	*pelX*
	pelX -R	CGGGGAGGAAAAATCGCCA	
	paeX -F	GTCTGTCAGTGGACTCAC	*paeX*
	paeX -R	TGTTCAAACAGATGGATTTC	
	paeY -F	CGATTACACTCAGACCATTC	*peaY*
	paeY -R	GATCGCGGTAATAAGGGTA	
	pemA -F	ACCATCAGCGCCAAGGATTT	*pemA*
	pemA -R	TACAGCGTGTCCTGATAGCC	
	pehK -F	CCATCTGGTGAGTTGCCGT	*pehK*
	pehK -R	TTTGGCGGTGGTTTTGATGC	
	pehN-F	AAGGTGTGGCGTTTTCGTTG	*pehN*
	pehN-R	AGCGCAATCTGCAAACGATG	
	pehX -F	GATTTACCAGAACGGCCAGC	*pehX*
	pehX -R	ACGGTGAACTGGTAGTCGGT	
	celZ -F	CACTCGCATTCAGCAGAAAACA	*celZ*
	celZ -R	AGTGCAATACCGTTATTTATCGC	

	celY -F	CAGCGCCGTGGTTTTGG	*celY*
	celY -R	GTGGCGGCAACTGATTGAC	
	bglA -F	TTTCGGCTGGATGTGTTCCG	*bglA*
	bglA -R	CCCACTTTGATATCCGGGTTGA	
	bgxA-F	GCGCGGCACCGGTTTT	*bgxA*
	bgxA-R	GGCGCGTTTGAAGCCCA	
	bglB -F	GGGTTCGGCACCATATTCA	*bglB*
	bglB -R	CGGAAGATGTGATGGAAAGC	
	nagZ -F	CCCGATGATAGAGCGTCTCC	*nagZ*
	nagZ -R	TCGGATGACCTGTCGATGG	
	celH –F	TCCACCACGCCGTTTTCATA	*celH*
	celH -R	TCTGGACCTGTGGTACAAGC	
	lfaA -F	GTTATAGCCCATCGCAT	*lfaA*
	lfaA -R	TGGGTATTGCACCATCA	
	prtX -F	ACATTGTAGTTGTACCAGGT	*petX*
	prtX -R	GGCGATACCGGGTTTG	
	prtC -F	TGCTCTGCATTAAATTTCACAA	*prtC*
	prtC -R	GTCAAGATGACGCTCAACAC	
	prtB -F	GGATAAGCCGCATAAGCCTG	*prtB*
	prtB -R	CCGCATACCACCTGGAAC	
	prtG -F	GCGTAGGTCACGTCTTTATAGC	*prtG*
	prtG -R	ATTGGAAGTGGGCCGGTT	
	prtD-F	ATCAACTGATCGATCGGGCT	*prtD*
	prtD -R	TACAGCCGTATTGCGTTGC	
	prtE -F	GCCTTCTTCGCTCACCTG	*prtE*
	prtE -R	ATGGGCGCATTCCGGT	
	prtF -F	CGGCCATCAGTTTGCG	*prtF*
	prtF -R	CATTCCAAGGTGACGCA	
	rhiE1-F	ATATGCGCTTCATGGTAACG	*rhiE1*
	rhiE1-R	GTACTACGCCCAGACTCAA	
	xynA-F	CGGATATACCACCAGACATA	*xynA*
	xynA-R	GAAGCCTGACTATGAATCCT	
